# Complete heart block and severe aortic stenosis in a patient with rheumatoid arthtritis: a case report

**DOI:** 10.1186/1757-1626-2-126

**Published:** 2009-02-05

**Authors:** Ioannis Moyssakis, Nikolaos Lionakis, Ioannis Vlahodimitris, Vassilios Votteas

**Affiliations:** 1 Laiko General Hospital of Athens, Department of Cardiology, 17 Agiou Thomas St, GR-15727 Goudi-Athens, Greece

## Abstract

**Background:**

A 77-year-old male patient with a history of rheumatoid arthritis was admitted to our hospital for investigation of syncope and dyspnea on exertion class II according to NYHA class association.

**Case presentation:**

The electrocardiogram revealed complete heart block whereas the echocardiogram showed severe aortic valve stenosis with a peak gradient = 80 mmHg. A permanent pacemaker was implanted in addition to aortic valve replacement. The coexistence of complete heart block and severe aortic stenosis with rheumatoid arthritis are presented.

**Conclusion:**

Further studies are necessary to assess whether a true association of the above conditions exist.

## Background

Rheumatoid arthritis is a chronic autoimmune multisystem disease which can affect the pericardium, myocardium, and endocardium. Heart involvement is a frequent cause of death [1-3].

This report present a case with severe aortic valve stenosis combined with complete heart block developing in a patient with rheumatoid arthritis. It would be interesting to see whether a linkage between the above cardiac lesions with rheumatoid arthritis can be demonstrated.

## Case report

A 77-year-old male with a known history of rheumatoid arthritis was admitted to our hospital due to syncopal episodes and dyspnea on exertion class II according to (NYHA). Rheumatoid arthritis was diagnosed 39 months ago on the basis of clinical and laboratory findings [4]. The patient was given corticosteroids (7.5 mg prednisone daily).

On physical examination her heart rate was 42 beats per minute and blood pressure 135/80 mmHg. Auscultation revealed a 3/6 systolic murmur at the right secont intercostals space which radiated to the carotids and significant diminished the second heart sound. Laboratory findings were as follows: hematocrit 34.9%, white cell count 8.800/mm^3^, platelet count 254000/mm^3^, erythrocyte sedimentation rate 57 mm/1^st ^h, CRP 10.5 mg/L urea 53 mg/dl, creatinine 1.1 mg/dl, SGOT 17, SGPT 24 U/L. Ra-test was negative whereas disease activity index and health assessment questionnaire score were 4.9 and 1,4 respectively.

**Figure 1:**
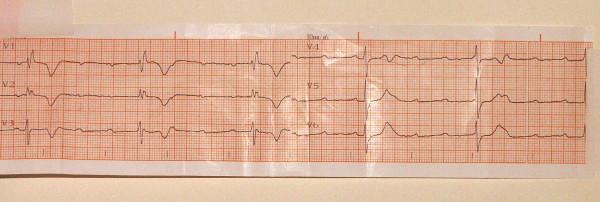
**The ECG of the patient showing the complete atrioventricular heart block**.

A resting electrocardiogram showed complete heart block (figure 1). Transthoracic echocardiography demonstrated severe left ventricular hypertrophy. The aortic valve was tricuspid with calcific and thickened aortic leaflets causing decreased opening. A transvalvular peak gradient of 80 mmHg was measured by continuous Doppler whereas a mild degree aortic regurgitation was revealed. Due to the fact that the ejection fraction was 45% the aortic valve orifice was measured which was 0.7 cm^2^. The patient sustained a coronary angiogram which was negative for coronary artery disease. Therefore, a permanent DDD pacemaker was implanted in addition to aortic valve replacement.

## Discussion

Rheumatoid arthritis is a common chronic autoimmune disease involving many organ systems, primarily the joints; frequently it is accompanied by cardiac lesions including pericardium, myocardium and endocardium, comprising coronary arteries, valvular tissue and conduction system. Heart involvement in RA is the leading cause of death [1-3].

Conduction disturbances are well recognized in the rheumatoid arthritis population. Primary infiltration by mononuclear cells or rheumatoid nodules could cause conduction disturbances at the sino-atrial or atrioventricular nodes or His-Purkinje system [5,6]. Other potential mechanisms are vasculitis of the arterial supply to conductive tissue, haemorrhage into a rheumatoid nodule or extension of an inflammatory lesion from the aortic or mitral valve [6]. Rarely, these lesions may be due to amyloid deposition [6]. Ahern et al have described complete heart block in 8 patients with rheumatoid arthritis and reviewed 20 similar cases previously reported.[7] However complete atrioventricular block is rare in RA and does not respond to anti-inflammatory and immunosuppressive therapy [6].

Valvular heart disease associated with rheumatoid arthritis has not been well characterized and its clinical predictors are undefined [8]. The incidence of rheumatoid valvular disease is among 2.5%–30% of necropsy patients with the above disease [9]. Valve nodules and valve thickening diffuse or localized by unspecific inflammatory changes were found in aortic and mitral valves [8,9]. No correlation was also found between valvular disease and duration, activity, severity, pattern of onset and course, extra-articular disease, serology, or therapy of RA [8].

Our case is interesting since there is no clear explanation for the severe valvular lesion combined with complete heart block in a patient with rheumatoid arthritis. In spite of the fact that the systemic disease was mild in severity complete heart block and severe aortic stenosis occurred. It should be noted that the patient had no history of arterial hypertension or renal involvement, conditions associated with aortic valve calcification. Moreover our case was not received anti-TNF and specifically infliximab therapy which are recognized causing atrioventricular block [10]. The existent severe aortic valve stenosis possibly could be associated with the atrioventricular block.

To our knowledge there are no reports in the medical literature of complete heart block and severe aortic valve stenosis in patients with rheumatoid arthritis. The association of the above cardiac lesions with rheumatoid arthritis may, however, be a random occurrence.

## Consent

Written informed consent was obtained from the patient for publication of this case report and any accompanying images. A copy of the written consent is available for review by the Editor in-Chief of this journal.

## Competing interests

The authors declare that they have no competing interests.

## Authors' contributions

IM conceived the case report. NL and IV were involved in the case management, and drafted the manuscript. IM, NL and VV reviewed the manuscript and made the final corrections before submission. All the authors have read and approved the final manuscript.
